# Somatic mutations in neurons during aging and neurodegeneration

**DOI:** 10.1007/s00401-018-1850-y

**Published:** 2018-04-28

**Authors:** Bert M. Verheijen, Marc Vermulst, Fred W. van Leeuwen

**Affiliations:** 1 Department of Translational Neuroscience, Brain Center Rudolf Magnus, University Medical Center Utrecht, 3584 CG Utrecht, The Netherlands; 2 Department of Neurology and Neurosurgery, Brain Center Rudolf Magnus, University Medical Center Utrecht, 3508 GA Utrecht, The Netherlands; 3 Department of Pathology and Laboratory Medicine, Children’s Hospital of Philadelphia, Philadelphia, PA 19104 USA; 4 Department of Neuroscience, Faculty of Health, Medicine and Life Sciences, Maastricht University, 6229 ER Maastricht, The Netherlands

**Keywords:** Somatic mutations, Genome integrity, Somatic brain mosaicism, Neuronal development, Aging, Neurological disorders, Neurodegeneration

## Abstract

The nervous system is composed of a large variety of neurons with a diverse array of morphological and functional properties. This heterogeneity is essential for the construction and maintenance of a distinct set of neural networks with unique characteristics. Accumulating evidence now indicates that neurons do not only differ at a functional level, but also at the genomic level. These genomic discrepancies seem to be the result of somatic mutations that emerge in nervous tissue during development and aging. Ultimately, these mutations bring about a genetically heterogeneous population of neurons, a phenomenon that is commonly referred to as “somatic brain mosaicism”. Improved understanding of the development and consequences of somatic brain mosaicism is crucial to understand the impact of somatic mutations on neuronal function in human aging and disease. Here, we highlight a number of topics related to somatic brain mosaicism, including some early experimental evidence for somatic mutations in post-mitotic neurons of the hypothalamo-neurohypophyseal system. We propose that age-related somatic mutations are particularly interesting, because aging is a major risk factor for a variety of neuronal diseases, including Alzheimer’s disease. We highlight potential links between somatic mutations and the development of these diseases and argue that recent advances in single-cell genomics and in vivo physiology have now finally made it possible to dissect the origins and consequences of neuronal mutations in unprecedented detail.

## Introduction

The vertebrate nervous system is a highly complex structure that is composed of many different cell types, including various types of neurons. This complexity enables specialized functions that are essential for proper organismal functioning. Although the physiological details that underlie this complexity remain unclear, it is at least partially mediated by the heterogeneity of neurons themselves, which can differ greatly in their morphology, as well as their connectivity and electrophysiological activity. Recent advances in single-cell expression profiling now allow us to explore this diversity further at the molecular level, and revealed that an even greater diversity of neuronal subtypes exists than previously expected, even within single brain regions composed of seemingly identical neurons [65, 66, 96, 150]. Although many studies using these experimental approaches are hampered by small sample sizes, a lack of time-points and inadequate assessment of distinct cellular states, it is anticipated that these technological innovations will ultimately lead to the identification of novel cell types, and contribute to a better systematic categorization of neurons [101]. In doing so, these experiments will greatly enhance our understanding of the origins and implications of cellular diversity within the nervous system [94] and elucidate how this diversity impacts neuronal function and disease.

**Fig. 1:**
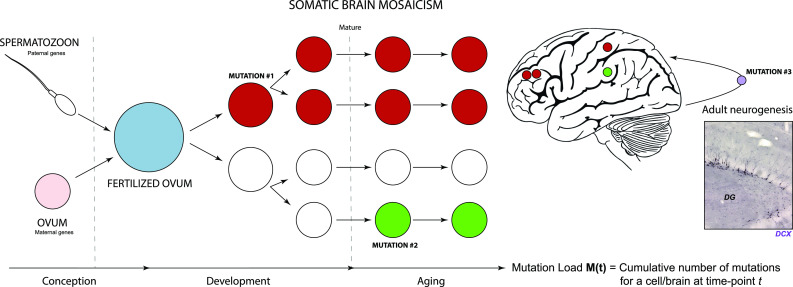
Somatic mutations in the nervous system. The genome is a set of instructions, or a program, for the development and functioning of organisms. It is often considered to be a fixed chemical entity, which is faithfully copied from mother to daughter cells during successive rounds of cell division and is mostly identical in different cells from different tissues. However, it has become clear that differences in genomes exist between single cells. Some researchers have taken the view that no cell in an individual does, in fact, carry the exact same genetic scripture. This potentially has major implications, especially for post-mitotic cells like neurons that are rarely or not at all replaced during life. Throughout normal development, post-zygotic mutations occur in neural progenitors, which are inherited by their cellular progeny (*mutation #1*). This will eventually culminate in a genetic mosaic. It has been suggested that somatic mutations can also take place in the developed nervous system (*mutation #2;* neurogenesis: *mutation #3*). Mature neurons are generally considered to be terminally differentiated post-mitotic cells with limited regenerative potential. Therefore, they are particularly prone to accumulation of damage. Specifically, genomic integrity of neurons can be influenced by the occurrence of gene mutations. During aging, the nervous system is subjected to various types of stress that contribute to neuronal damage, including genomic alterations such as telomere shortening and chromosomal abnormalities. These changes are accompanied by various other alterations, like impaired nuclear integrity, aberrant nucleocytoplasmic transport and defects at the level of mitochondria (including mtDNA mutations). Insert shows a microscopic image of the dentate gyrus of a mouse hippocampus, immunostained for doublecortin (DCX), a marker for neurogenesis (Verheijen, Vermulst and van Leeuwen, unpublished)

In addition to tightly orchestrated programs that differentiate neurons into highly specialized subtypes, a growing body of evidence indicates that neurons can also differ at the genomic level [8, 42]. During neuronal development, somatic mutations may arise in neuronal stem/progenitor cells that are propagated throughout the neural lineage these cells spawn. This propagation leads to the formation of a “mosaic patchwork” of genetically different neurons, a phenomenon that is commonly referred to as “somatic brain mosaicism” (Fig. 1). By altering the genetic makeup of cells, these mutations could perturb critical aspects of neuronal function and contribute to various neurological diseases [70, 85, 98, 100]. For example, neuronal mutations could affect genetic programs that define neuronal identity or alter properties that are essential to the development and plasticity of neural circuits, such as the organization of dendritic spines and axonal boutons, or the efficiency of synaptic transmission. Similar changes could affect the selective vulnerability of neuronal cells to insults and disease, and contribute to complex quantitative phenotypes, such as the genetic architecture of behavior and cognition. Moreover, randomly generated genetic diversity within the brain might, at least in part, explain personal differences between otherwise identical individuals [24]. It is compelling that the existence of a general “proantigen” that undergoes minor genomic changes during brain development, giving rise to a variety of “neurotypes”, has already been proposed several decades ago [123].

However, age-related mutations in post-mitotic neurons, accumulating over the course of human aging, represent a relatively poorly studied group of mutations. They are of exceptional interest though, because aging is a major risk factor for many neurological diseases, including Alzheimer’s disease (AD). Insights into the mechanisms and consequences of neuronal mutations could therefore lead to a better understanding of the etiology of disease progression and hint at novel strategies for therapeutic intervention. Intriguingly, this insight may even provide us with a better understanding of the aging process of the brain itself [60, 62, 77, 127].

In the present review, we highlight some of the early work on somatic mutations in the hypothalamo-neurohypophyseal system, which provided the first experimental evidence for somatic mutagenesis in aging neurons. Next, we delve into the mechanisms that could be responsible for somatic brain mosaicism and discuss the potential contribution of neuronal somatic mutations during development and aging to neuropathology. Finally, we present a road map to guide continued efforts to elucidate the somatic mutation burden in brain tissue and to better understand their impact on human aging and disease.

## Somatic mutations in vasopressin neurons

**Fig. 2:**
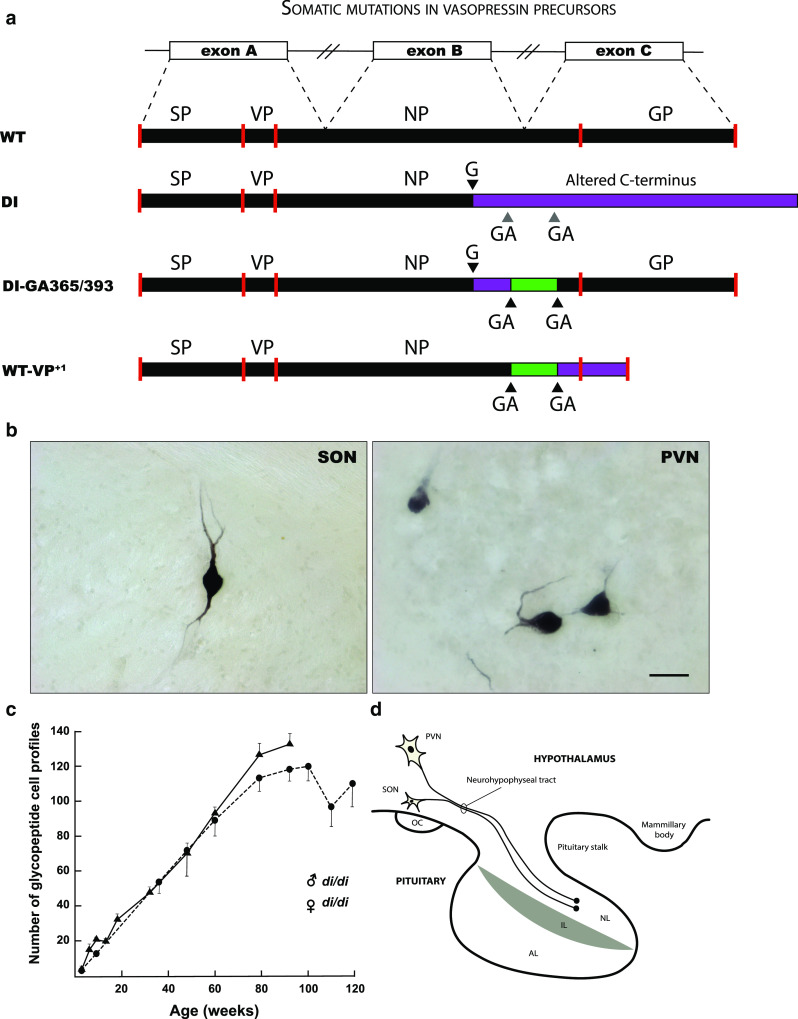
Mutations in post-mitotic vasopressin neurons. **a** Schematic representation of the vasopressin (*VP*) gene, VP prohormone and mutant forms. The *VP* gene consists of three exons (exon A, B, and C, consisting of 429 nucleotides), which give rise to a transcript that is spliced to generate the mRNA template for a precursor protein (*WT*). VP precursor protein is translated in the endoplasmatic reticulum (ER), post-translationally processed and packaged within neurosecretory granules. Subsequently, the protein is axonally transported to nerve terminals in the neural lobe of the pituitary gland (**d**). In the homozygous (di/di) Brattleboro rat, a single base (G) deletion in exon B results in an out-of-frame protein that contains a poly-lysine tail that cannot be properly processed (*DI*). This results in (central) diabetes insipidus (DI), a condition that is characterized by polyuria and polydipsia, due to the inability to effectively regulate the VP-mediated retention of water in the kidney’s collection ducts (antidiuretic function). This is an autosomal recessive trait that is inherited in a simple Mendelian fashion. **b** Intriguingly, some solitary neurons in the supraoptic nucleus (SON) and paraventricular nucleus (PVN) of the homozygous Brattleboro rat appear to be immunoreactive for VP. Bar = 50 μm (Verheijen, Vermulst and van Leeuwen, unpublished). This appears to be due to a second mutation (ΔGA) in a GAGAG motif that is located downstream of the single base mutation (**a**). As a result the VP mutant precursor (*DI*-*GA365/393*) can be processed (i.e., the glycoprotein (GP)-containing part) and the neurosecretory granules can be axonally transported towards the neural lobe. The amount of *reverted* neurons (+/di) increases age-dependently in both male (filled triangle) and female (filled circle) rats (**c**). Because GAGAG motifs are also present in the wild-type *VP* gene of rat and human, a similar process can take place and convert the wild-type VP precursor into an aberrant one (*WT*-*VP*^+*1*^). This has been shown to occur in hypothalamus of both rat and human. *AL* anterior lobe, *IL* intermediate lobe, *NL* neural lobe, *NP* neurophysin, *OC* optic chiasm, *SP* signal peptide

Some of the earliest experimental evidence for mutations in post-mitotic neurons came from observations in the homozygous (di/di) Brattleboro rat, which carries a naturally occurring mutation in the vasopressin gene (*VP*) [108, 132]. The di/di rat suffers from severe diabetes insipidus due to a single base deletion in exon B of the *VP* gene, resulting in an abnormal VP precursor frameshift mutant (Fig. 2a). This frameshift mutant cannot translocate in the endoplasmatic reticulum (ER) and, therefore, cannot be axonally transported towards the neural lobe of the neurohypophysis where it is secreted into the circulation (Fig. 2d).

Surprisingly, immunocytochemistry revealed that some solitary neurons in the hypothalamus of the KO rat are reactive for the VP precursor, as shown by immunoreactivity for its C-terminal glycopeptide (GP) domain (Fig. 2b) [132]. This finding indicated the occurrence of a post-mitotic mutation event (+/di) in these cells. Interestingly, the number of GP-positive neurons increases over age, suggesting an age-acquired *cell reversal* phenotype (Fig. 2c) [132, 135]. This age-dependent increase in reverted cells is remarkably linear, indicating a fixed mutation rate (approx. 1 cell/week). Through this work, similar post-mitotic mutations were found in wild-type rats and in hypothalamic neurons in human brain [39, 41]. Of note, a similar age-related reversal phenomenon has been described to occur in liver of analbuminemic rats (for a detailed overview, see [46]).

It has later been proposed that the observed *VP* mutations occur in certain repeat motifs (e.g., GAGAG motifs) at the RNA level, representing a specific type of transcription error [19, 133]. Certain regions in the genome may be particularly prone to transcription errors due to polymerase slippage/stuttering or other mechanisms. A comprehensive overview of the origins and consequences of errors in neuronal gene expression and its potential relation to VP is beyond the scope of this review (but see—Future directions—“Beyond the genome: neuronal epimutations”), but the notion of transcript mutations in di/di rats has been challenged by the observation that: (i) the reversal is an all or nothing event. In other words, only high levels of altered transcripts are present in select neurosecretory neurons or none at all, which is inconsistent with the presence of a transcript mutation; (ii) there is a constant increase in the number of reverted cells with age, suggesting an irreversible phenotype [40]. However, we cannot rule out the existence of some kind of RNA editing event causing this cell reversal phenotype. Notably, somatic DNA mutations may cause shifts in the accuracy of gene expression or affect control over genomic integrity itself (e.g., by affecting cellular machinery involved in regulating gene expression and cellular quality control mechanisms). Integrated DNA-RNA sequencing, i.e., DNA *and* RNA sequencing of the same reverted cells, could provide a definitive explanation for this phenomenon. Solitary VP neurons can be easily dissected under the microscope. If these experiments confirm that cellular reversal of the *VP* gene is caused by a genetic mutation, the di/di rat might be a useful model system to study mechanisms of somatic mutation in the adult nervous system.

Since these early observations on the Brattleboro rats, reporter mice and highly sensitive single-cell sequencing techniques have been developed that have recently confirmed the existence of genetic mutations in post-mitotic neurons [9, 75]. These studies now indicate that genetic mutations do not only occur in the *VP* gene, but are also present throughout the genome of neuronal cells. As a result, these mutations can affect a wide variety of biological processes, which could affect the architecture of the brain and behavior in numerous ways. However, how these somatic mutations arise remains heavily debated.

## Mechanisms for somatic mutations in neurons

**Fig. 3:**
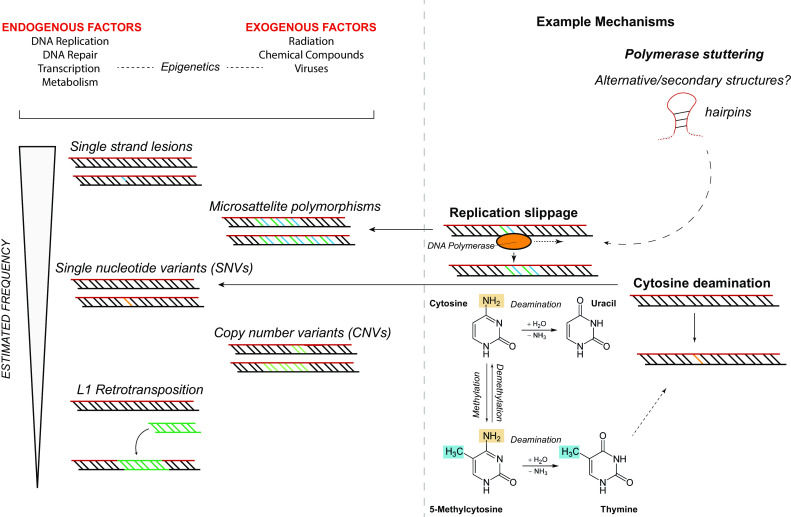
Various types and mechanisms of neuronal somatic mutations. Different types of mutations have been found to be present in single neurons, including long interspersed nuclear element 1 (L1 or LINE1) retrotransposition, copy-number variations (CNVs), single-nucleotide variants (SNVs), and microsatellite/short tandem repeat variants. The exact contribution of each of these events to somatic neuronal mosaicism is unknown. Also, the mechanisms through which these mutations can arise are mostly unknown. Slippage of DNA polymerases, e.g., due to secondary structures in the chromatin, can cause changes in length of microsatellites. Cytosine deamination has been recognized as a frequent cause of SNVs. It will be necessary to accurately quantify these different types of mutations, for various cell types and brain regions, during different developmental stages or under particular conditions, in order to gain insight into their (potential) roles

In the developing nervous system, replication errors during S-phase are the best-known source of neuronal mutations. The rapidly proliferating neural progenitors that are responsible for populating the developing brain are exquisitely sensitive to errors that occur during DNA replication and chromosome segregation, two potent sources of de novo mutations. Interestingly, long neural genes are particularly prone to DNA breaks, which provides an intriguing explanation for a fraction of the observed variety in brain DNA [143, 144]. However, in the adult nervous system, which is primarily composed of post-mitotic cells that do not undergo DNA replication, replication errors cannot explain genomic differences (with the exception of cells that arise in the neurogenic niche of the subventricular zone and the dentate gyrus) [15, 36]. A fascinating relationship between attempted cell cycle re-entry (G_0_ → S) and neurodegeneration has recently drawn some attention though [27, 148]. Accordingly, both neurogenesis and early embryogenesis exhibit a drastic increase in mutagenesis compared to adulthood [9]. This observation suggests that other endogenous (spontaneous) and exogenous (environmental) factors must be responsible for the accumulation of mutations in post-mitotic cells (Fig. 3) [81].

Exogenous factors that are well known to induce mutagenesis include environmental stressors such as electromagnetic radiation, viruses, and mutagenic chemicals. A lifetime of exposure to these genotoxins is likely to induce mutations in aging neurons, although it should be noted that intra-uterine exposure to these toxins (e.g., through maternal smoking) could also introduce mutations in neurons during development [99].

Endogenously, multiple mechanisms contribute to mutational events. These include DNA damage, DNA replication, defective DNA damage repair, transcription, and metabolic processes (e.g., through the generation of reactive oxygen species). Of these various processes DNA damage is an especially powerful source of somatic mutations. For example, single-base substitutions can be introduced by cytosine deamination events, a highly mutagenic lesion that can potentially result in transition mutations [63].

In the context of DNA damage, it is interesting to note that there might be an underappreciated role for neuronal activity in the generation of DNA damage. Physiological neuronal activity has been shown to induce double-strand breaks in DNA [124]. The VP neuron reversal events in di/di rats mentioned in the previous section (“Somatic mutations in vasopressin neurons”) appear to be related to activity status as well [40, 41]. It would be interesting to explore whether a relationship exists between neuronal activity, plasticity and memory, and plasticity at the level of the genome. Excitingly, it was recently shown that early life experiences can indeed influence DNA in the brains of rodents [12, 120].

Regardless, given the role that DNA damage plays in mutation accumulation, DNA repair must be a powerful modifier of mutagenesis as well [83, 87, 88, 105, 115]. It is conceivable for example, that an age-related loss of DNA repair efficiency (potentially due to mutations in genes encoding DNA repair proteins) could contribute to an increase in genomic instability during aging [27]. It should be noted though that mutations also occur on pristine DNA templates. For example, differences in microsatellite repeat length (a highly prevalent somatic mutation) can be caused by DNA polymerase slippage during replication. Similar replication slippage events occur on poly(A) tracts after somatic long interspersed nuclear element 1 (L1) retrotransposition events.

Mutations may also arise due to unexpected events. For example, proteins that are part of the V(D)J site-specific recombination machinery, such as recombination activating gene-1 (RAG-1), have long been known to be present in neurons [28]. This observation raises the possibility that similarities may exist between the mechanism of recombination and somatic hypermutation in B cells of the immune system and somatic mutations in neurons [33, 40]. How these mutations might affect cellular function is largely unknown. It is likely though, that because each type of mutation has a unique signature, individual mutations may affect neuronal function in unique ways. For example, copy-number variations (CNVs) can result in changes in protein synthesis [23], and endogenous retroelements (such as L1) can disrupt genes or gene regulatory elements [10, 29, 37, 43, 45, 93, 117, 129]. These alterations in gene expression could have very pronounced effects on neuronal function, e.g. via transdifferentiation of cells to other phenotypes (phenotypic switching) or by altering the wiring of the nervous system. As a result, somatic mutations could progressively alter the behavior of individual neurons and the neural networks they support. For example, the slow accumulation of mutations over time could explain why the first signs of neurodegeneration appear decades before behavioral signs of AD pathology appear [16]. However, the impact of post-mitotic mutations on diverse neuronal subtypes may be greater than expected based on genes alone: distinct types of neurons show differential gene expression, expression of noncoding elements, patterns of splicing and post-translational modifications of their proteins. Therefore, similar post-mitotic mutation events, potentially interacting with every aspect of cell biology, may have very broad phenotypic consequences in different neurons.

Accurate assessment of somatic mutation accumulation rates in different cell types, tissues, brain regions and species, under different conditions, remains a major challenge in the field. Different aspects of sequencing approaches, bioinformatic analyses, and validation methods can lead to artifacts that are erroneously identified as somatic mutation events [44, 70, 131, 145]. Therefore, it can be difficult to accurately calculate the mutation load of human neurons. However, a reasonable estimate of the mutation frequency in the healthy human brain suggests though that single neurons harbor approximately 800–2000 single-nucleotide variants (SNVs); 80% of these SNVs are C > T transitions [76]. In addition, approximately 13–41% of human frontal cortex neurons contain at least one megabase-sized de novo CNV [84]. Technical improvements will undoubtedly contribute to improved estimates of mutation rates, assist in filtering out false positives/noise from genuine somatic mutations, and thereby result in new insights into their etiology and importance.

## Somatic mutations in neurological disorders

Somatic mutations in neurons have been demonstrated to be involved in several neuronal (developmental) disorders. For example, cancers of the nervous system have long been associated with somatic mutations. However, it is now clear that the link between somatic mutations and disease is not limited to cancer [20, 70, 79, 85, 100, 116].

An example of a neurodevelopmental disorder that can be caused by somatic mutations is focal cortical dysplasia (FCD), which is a cause of epilepsy. Mutations in components of the mammalian target of rapamycin (mTOR) pathway are one cause of FCD, and mTOR pathway-related changes in brain (micro)circuitry are likely to cause seizures [31, 73]. Of course, somatic mutations and somatic brain mosaicism could cause many other neurological disorders as well, or predispose individuals to the development of these diseases. Key examples include developmental disorders such as autism [85], or diseases with an age-related component, such as AD [6, 11, 21, 51, 92, 111], Parkinson’s disease [102, 103], prion disease [3], and amyotrophic lateral sclerosis (ALS) [69, 86] (Table 1). Strikingly, somatic mutations do not even need to occur in neuronal cells to cause neuropathology [82]. Similar to cancer, it has been speculated that the occurrence of these age-related neurological disorders may be explained by a “multiple-hit” model, a combination of developmental predisposition and somatic mutagenesis. Somatic mosaicism and age-related somatic mutations could explain for at least a number of (early-onset) sporadic (non-familial) cases [22]. However, this hypothesis could also apply to a number of other neurological disorders, like schizophrenia, semantic dementia, and a broad range of neurological phenotypes that are characterized by intellectual disability. Intriguingly, the random nature of somatic mosaicism could also be a factor explaining disease discordance in monozygotic twins [13].

**Table 1:** Evidence for somatic mutations in neurodegenerative disease

Disease	*N* (cases)	Gene(s)	Type of mutation	Approximate level (%)	Approach	References
AD	1	*PS1*	SNV	14	PCR, ASOH, Sanger sequencing	[11]
	32	*APP*	CNV	[single-cell]	Single-cell qPCR, FISH	[21]
	72	*APP*, *PS1*, *PS2*, *MAPT*	CNV, SNV	≥ 10 (CNV), ≥ 1 (SNV)	Targeted deep sequencing, qPCR	[111]
PD/DLB	567	*SNCA*	SNV	≥ 5–10	PCR (HRM)	[102, 103]
Prion disease	1	*PRNP*	SNV	97	PCR, Sanger sequencing, qPCR	[3]

*AD* Alzheimer’s disease, *APP* amyloid precursor protein, *ASOH* allele-specific oligonucleotide hybridization, *CNV* copy-number variations, *DLB* dementia with Lewy bodies, *FISH* fluorescent in situ hybridization, *HRM* high resolution melt analysis, *MAPT* tau protein, *PCR* polymerase chain reaction, *PD* Parkinson’s disease, *PRNP* prion protein, *PS1* presenilin 1, *PS2* presenilin 2, *qPCR* quantitative PCR, *SNCA* alpha-synuclein, *SNV* single-nucleotide variant

Partially based on [70]

**Fig. 4:**
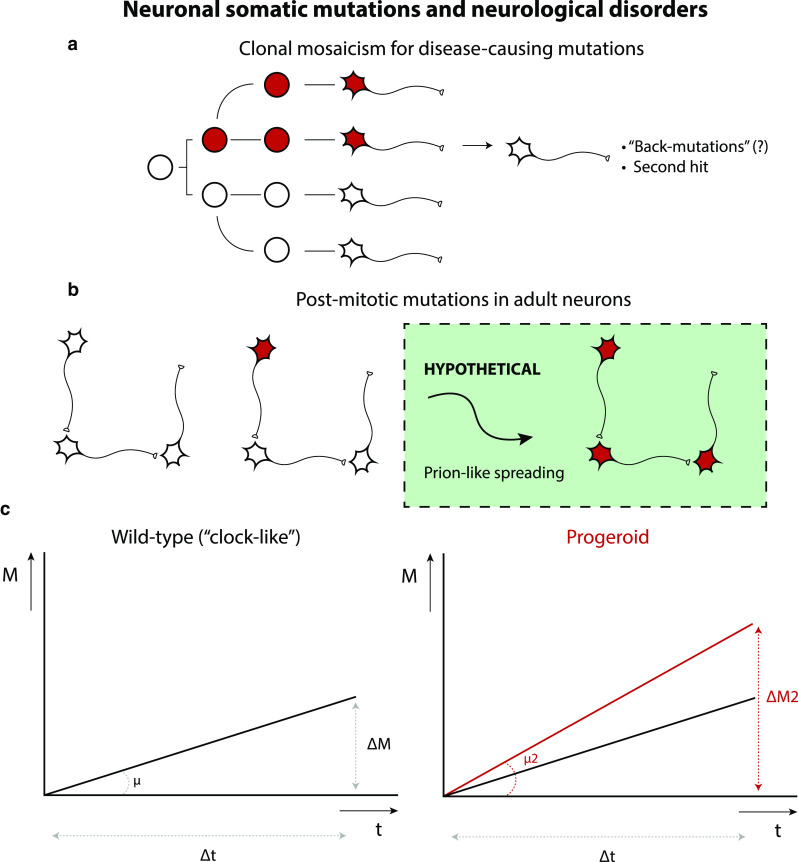
Neuronal somatic mutations and neurological disorders. Somatic mutations in neurons could cause or predispose for neuronal diseases. Neuronal somatic mutations can either occur in neuronal progenitors, giving rise to mutant daughter cells through clonal expansion of these mutation-carrying cells (**a**) or in post-mitotic neurons (**b**), resulting in very fine changes in the nervous system. A combination of both might reflect an intriguing mechanistic link between developmental and degenerative brain disorders. Neuron-to-neuron spreading of pathological proteins could provide an explanation for widespread pathology induced by single-neuron DNA mutations, but this model is purely hypothetical as of now. **c** Somatic mutations have been found to accumulate in neurons as a function of age. Neuronal somatic mutation accumulation could be a general hallmark of neuronal aging. In DNA repair disorders (Cockayne syndrome, Xeroderma pigmentosum) increased rates of somatic mutations (*μ*) have been observed (“progeroid” phenotype). Increased rates of somatic mutation in single neurons could be important for other neurodegenerative disorders and may also accelerate neuronal aging itself

As pointed out before, there is a fundamental difference between the clonal somatic mosaicism that is generated during neuronal development (Fig. 4a) and somatic mutations that arise in post-mitotic neurons (Fig. 4b).

Interestingly, in mosaic Down syndrome, wherein only some cells show trisomy 21, the degree of mosaicism (percentage of trisomic cells) is associated with disease severity [97]. The same holds true for other diseases with large structural abnormalities (e.g., aneuploidy, chromothripsis). Though it may seem strange, normal brains contain a certain amount of “constitutive” aneuploidy [61, 107]. Genomic duplication can also cause AD [110, 119]. For post-mitotic mutations that initiate disease, it is to be expected that a certain cascade of events occurs that ultimately results in pathology. For example, it is conceivable that some mutations in single neurons could give rise to aberrant proteins with prion-like properties that spread to other areas of the nervous system to induce disease there (“seeding”) [1, 18]. Although the concept of somatic mutation-induced prion-like spreading is purely hypothetical as of now, the potential implications are enormous: elegant transplantation experiments of human stem cell-derived cortical neuronal precursors into brain of murine AD model induced hallmarks of AD in these human neurons, consistent with disease spread via neighboring cells [38].

In a recent single-cell whole-genome sequencing study, different somatic single-nucleotide variant (sSNV) mutational signatures were identified in single post-mitotic neurons [75]. sSNVs were found to increase approximately linearly with age (“*genosenium*”), with different mutation rates in different brain areas, and were more abundant (up to ~ 2.5-fold higher) in early-onset neurodegenerative diseases caused by defective DNA repair (i.e., Cockayne syndrome or Xeroderma pigmentosum, diseases that are characterized by defective nucleotide excision repair) (Fig. 4c). This is an interesting observation, because mutations in some other neurodegenerative diseases, such as ALS, are known to affect DNA repair as well [142]. Another interesting possibility is that disease-associated pathogenic proteins may promote mutations in neurons overall [8, 124], or could awaken dormant genetic elements with pathogenic properties [71].

Specifically, at least 3 different mutational signatures have been identified in brain [75]:

- *Signature A, or a* “*clock*-*like*” signature [2], composed mostly of C > T and T > C mutations and the only signature to increase with age, regardless of brain region or disease state.
- *Signature B*, a “*neurodevelopment*” signature, consisting primarily of C > T mutations that do not correlate with age, hinting at a mutational mechanism at early ages, perhaps prenatally, and associated with neurogenesis.
- *Signature C*, a “*disease*” signature, distinguished by the presence of C > A variants, mutations that are closely associated with oxidative DNA damage, and strongly associated disease states (i.e., neurons defective in DNA damage repair).

The existence of a “clock-like” mutational signature that shows a linear increase of somatic mutation accumulation in neurons is of particular interest, because somatic mutation accumulation/genomic instability has been suggested as a mechanism of (neuronal) aging itself [27, 126, 141]. Interestingly, C > T mutations are the most abundant substitutions induced by oxidative DNA damage. Typically, these mutations are caused by oxidized cytosines that are initially converted into 5-hydroxycytosine and then metabolized further into 5-hydroxyuracil and uracil glycol [63]. Possibly then, this signature is caused by the highly metabolic nature of neurons, and the reactive oxygen species that tend to leak out of mitochondria. Accordingly, overexpression of a mitochondrially targeted catalase prevents the accumulation of C > T mutations in the mitochondrial DNA of aging mouse brains [139]. In combination with signature C, these observations suggest that oxidative damage is a primary cause of somatic mutations in neuronal cells. As a result, it might be interesting to measure somatic mutation load in cohorts that appear to age differently from the general population, such as (super)centenarians [49], and to monitor their anti-oxidant defenses. The potential significance of neuronal somatic mutations to disease, perhaps contributing to all sporadic disease cases, will be a strong incentive for future studies to explore their occurrence and phenotypic effects in more detail.

## Future directions

### Technical aspects

Despite a number of conceptual and technological breakthroughs, the presence of somatic mutations in neurons remains an enigmatic phenomenon. To solve this riddle, future research should focus on developing an even greater catalogue of somatic mutations in adult neurons. Early experimental models, such as the LacZ-plasmid reporter mouse model (or: “in vivo Ames test”) introduced by Vijg et al., provided important insights into mutations in single cells [14, 54]. However, such reporter loci cannot be used as a representative measure for spontaneous mutation rates in the *whole* genome. To study somatic mutagenesis throughout the genome, it will be critical to select the correct next-gen sequencing techniques. Recently, deep exome sequencing of DNA derived from bulk brain tissue has emerged as a valid technique for detecting somatic mutations, because it minimizes sequencing artifacts that confound downstream analyses. However, we expect that so-called duplex sequencing technology holds the greatest promise for the detection of somatic mutations in human tissues [56, 112]. This technique hinges on a clever barcoding strategy that circumvents most of the problems that plague mutation discovery to this day. An important drawback of both of these sequencing techniques though is that somatic mutations that occur in post-mitotic neurons are specific to single cells and can therefore only be conclusively identified through single-cell genome comparisons [34, 75, 85]. Therefore, a combination of single cell and next-gen sequencing technology may be required to solve these problems definitively. We expect that by leveraging this new technology, and further developing existing techniques, mutational patterns will undoubtedly emerge that will help explain the molecular mechanisms that underlie somatic brain mosaicism. Accurate mapping of somatic mutations in single neurons is technically challenging though, and it is therefore likely that technology development will remain a very important goal of the field in the foreseeable future. For example, the low amounts of genomic material that can be collected from individual cells require an initial amplification-step. In vitro nucleic acid amplification methods such as PCR are known to be error-prone, which could contribute to a faulty interpretation of sequencing results. An interesting solution to this problem is the transplantation of nuclei from post-mitotic olfactory bulb neurons to enucleated oocytes, which allows the endogenous replication machinery to amplify the genetic material. Through this method, hundreds of mutations were recently identified in clonally expanded neurons from mice [58]. Although this approach may provide a more precise estimation of the mutational profile in these cells, it is limited by its technically challenging and time-consuming nature. Thus, further improvements in single-cell sequencing techniques [50] would greatly benefit our understanding of somatic brain mosaicism.

Ideally, these improvements would be coupled to advanced microscopy techniques that can delineate the architecture of the brain in unprecedented detail [4], in combination with better tissue clearing [5, 109] so that the structural and temporal aspects of brain organization can be compared directly to the timing and development of somatic brain mosaicism. The use of advanced microscopy techniques would also limit confusion caused by sampling variations in the current literature, by allowing researchers from different labs to sample the exact same cells from the exact same brain areas with a greater degree of accuracy. The improved accuracy of these experiments would also allow a greater number of cells to be collected and analyzed, further removing additional sampling biases. And finally, by improving the bioinformatic pipelines that are used for the analysis of these cells, it will be possible to better interpret these complex datasets [48, 75, 85].

**Fig. 5:**
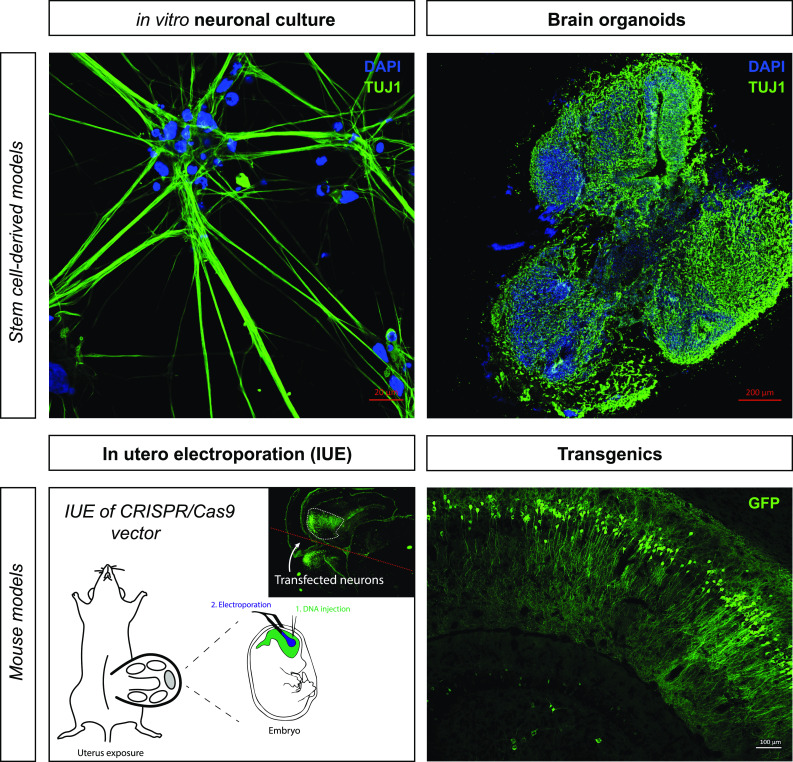
Experimental strategies to model brain somatic mosaicism. Different experimental model systems can be used to mimic somatic mosaicism in the nervous system. For in vitro study of neuronal somatic mutations, hiPSC-derived neurons would be an attractive option, because they are human cells and can be derived from little starting material (through clonal expansion). Brain organoids are 3-dimensional models for neuronal brain development and disease. These also allow grafting of single neurons and grafting into live brains or other parts of organoids (e.g., to study mechanisms of prion-like spreading). Manipulated organoids can be optically cleared and imaged in 3D, e.g., using light sheet microscopy. Depending on the biological questions being posed, and especially the window of time and spatial requirements, different approaches can be considered. To study the in vivo contribution of somatic mutations, gene-editing constructs can be transfected in developing mouse brain via in utero electroporation of embryonic mice. Electroporation of a mouse brain (or organoid) will result in mosaic expression by default, because only select cells are transfected. In the adult brain, delivery of gene-editing ribonucleoprotein complexes (RNPs) or viral agents can be considered. Alternatively, (inducible) transgenic mice can be used to generate mosaics (Verheijen, Vermulst and van Leeuwen, unpublished)

However, sequencing is only a starting point for new experiments [17, 106]. In the future, it will be equally important to elucidate the downstream effects of somatic mutations on neuronal function. For example, in situ gene editing, via in utero electroporation of gene editing constructs or local delivery of engineered ribonucleoprotein complexes [72, 85, 122] could be a powerful method to unravel the functional consequences of genomic variability in neurons. The search for phenotypes will surely be aided by improvements in in vivo physiology approaches. We anticipate that smaller animal models will also be a powerful tool to understand the impact of somatic mutations on neuronal function. For example, exploring the effects of neuronal somatic mutations on the nervous system of *C. elegans* would be especially exciting, because the wiring diagram of the nervous system of these worms is well-characterized. Human experimental models, such as stem cell-derived neurons and cerebral organoids, would also be a valuable tool to better understand the non-uniform genomic organization of the nervous system [7, 26, 32, 67, 68, 80, 104, 113] (Fig. 5). In vitro aging of single neurons and modeling neurodevelopment in 3-dimensional cultures will enable exquisite control over experimental conditions. It would be very interesting to measure the somatic mutation load in different types of organoid cultures, to compare tissue-type specific mutation rates [59] and species differences [90]. Neural circuits disrupted by somatic mutation can be further studied experimentally using a battery of tests, including (2-photon) calcium imaging [147] and multi-electrode arrays [121] or optogenetic approaches [89, 118], to analyze networks. Transcriptomics, proteomics, and metabolic profiling can be used to further explore the effects of mutations on gene expression and metabolite fingerprints, but particular phenotypes associated with specific mutations and diseases will probably require specialized (biochemical) assays.

Finally, it will also be important to carry out neuropathological examinations to validate findings related to brain mosaicism. (Single-cell) DNA sequencing will be the main approach to identify somatic mutations in neurons, but checking these findings against the reality of human tissues will be crucial to avoid errors introduced by technical artifacts. In situ hybridization using probes that specifically recognize abnormal gene products would be a straightforward method to look for abnormalities in single cells. Antibodies that specifically recognize certain mutant proteins have also been utilized for this purpose in the past [39, 134]. Combining this information with other neuropathological indicators, like abnormal cellular morphology and cellular stress markers, could also be a means to link somatic mutations to disease processes.

### Beyond the genome: neuronal epimutations

Until now, most of the work on somatic brain mosaicism has focused on genetic changes in DNA (including mitochondrial DNA [25]). It is possible though that non-genetic changes play a role in brain mosaicism as well. After all, the damage that is thought to be responsible for the accumulation of genetic mutations in aging neurons (e.g., oxidative and mechanical damage) also affects other cellular processes. For example, certain DNA lesions reduce the fidelity of RNA polymerase II, which provokes transcription errors, or so-called epimutations, in neuronal cells [114]. It is now clear that despite their transient nature, these transcriptional noise events may result in robust phenotypic changes and disease [53, 55, 138, 140].

**Fig. 6:**
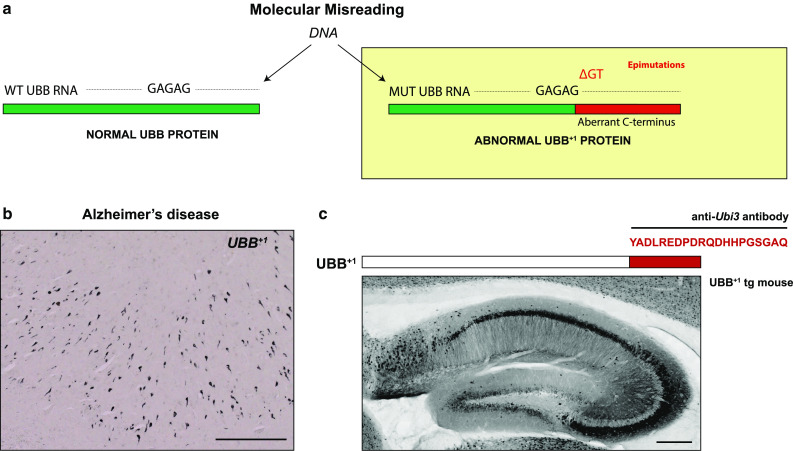
Neuronal epimutations. **a** Schematic representation of “molecular misreading”, a form of transcriptional mutagenesis or “epimutation”. (Epi)genomic drift and an overall increase in transcriptional noise may contribute to age-related neurodegenerative processes. Dinucleotide deletions (e.g., ΔGA or ΔGU) in an mRNA molecule can result in the generation of mutant proteins, which can be recognized by specific antibodies. **b** An example of such a mutant protein is ubiquitin-B^+1^ (UBB^+1^). UBB^+1^ is detectable in the brains of Alzheimer’s disease (AD) patient brains, by immunocytochemistry using antibodies that specifically recognize the abnormal C-terminal domain of UBB^+1^. This demonstrates the importance of neuropathological observation in validating these mutational events. Scale bar: 200 μm. **c** Transgenic expression of UBB^+1^ in mice results in behavioral phenotypes that are consistent with neurodegeneration. Image shows a sagittal section of the hippocampus of a UBB^+1^ transgenic mouse, stained for UBB^+1^. Scale bar: 200 μm (Verheijen, Vermulst and van Leeuwen, unpublished)

For example, through a mechanism dubbed “molecular misreading”, which describes the inaccurate conversion of DNA to RNA during transcription, frameshift mutants can arise of the ubiquitin-B (UBB) protein (i.e., UBB^+1^) [130, 133, 134, 136, 137] (Fig. 6). Importantly, UBB^+1^ is an inhibitor of the ubiquitin proteasome system (UPS) and delays the targeted destruction of both normal and abnormal proteins, thereby compromising the integrity of the proteome. Interestingly, UBB^+1^ accumulates in the neuropathological hallmarks of Alzheimer’s disease (AD) and several other disorders [52], and by inhibiting the UPS, UBB^+1^ is thought to promote the aggregation of toxic peptides that play a role in disease progression.

Programs for the modification of DNA and 3D chromatin [47, 64, 78, 91, 95, 128], RNA and proteins, such as RNA modification [149], protein conformational conversion [57], and post-translational modifications add another layer of complexity to these phenomena. For example, RNA editing is specifically enriched in the nervous system of behaviorally sophisticated coleoid cephalopods, affecting molecules that are essential for the morphology and excitability of neuronal cells [74]. With the introduction of new RNA-targeting tools [146, 30], it may now be possible to study these events in unprecedented detail. However, we are only beginning to explore the origins and downstream consequences of non-genetic mutations in the nervous system. But with improved detection techniques, and a better understanding of their physiological consequences, these observations could greatly enrich our understanding of somatic brain mosaicisms (as well as mosaicisms in other tissues [35, 125]) and ultimately contribute to better diagnoses and treatments with next generation, personalized drugs.
