# Correction to: Somatic mutations in neurons during aging and neurodegeneration

**DOI:** 10.1007/s00401-020-02186-y

**Published:** 2020-07-06

**Authors:** Bert M. Verheijen, Marc Vermulst, Fred W. van Leeuwen

**Affiliations:** 1 Department of Translational Neuroscience, Brain Center Rudolf Magnus, University Medical Center Utrecht, 3584 CG Utrecht, The Netherlands; 2 Department of Neurology and Neurosurgery, Brain Center Rudolf Magnus, University Medical Center Utrecht, 3508 GA Utrecht, The Netherlands; 3 Department of Pathology and Laboratory Medicine, Children’s Hospital of Philadelphia, Philadelphia, PA 19104 USA; 4 Department of Neuroscience, Faculty of Health, Medicine and Life Sciences, Maastricht University, 6229 ER Maastricht, The Netherlands

## Correction to: Acta Neuropathologica (2018) 135:811–826 10.1007/s00401-018-1850-y

In the original article, the panels “Brain organoids” and “Transgenics” were included in Fig. 5 without permission. Additionally, the statements “Verheijen, Vermulst and van Leeuwen, unpublished” in the Fig. 5 legend and “Images in Fig. 5 were obtained by B.M.V. in the lab of Prof. Dr. R.J. Pasterkamp (MIND Facility, Brain Center Rudolf Magnus, Utrecht, The Netherlands)” should not have been included. The authors apologize for the error and state that it does not change the scientific conclusions of the article.

